# Moderating Effects of Early Pointing on Developmental Trajectories of Word Comprehension and Production

**DOI:** 10.3390/ijerph19042199

**Published:** 2022-02-15

**Authors:** Paola Perucchini, Arianna Bello, Fabio Presaghi, Tiziana Aureli

**Affiliations:** 1 Department of Education, Roma Tre University, Via Castro Pretorio 20, 00185 Roma, Italy; arianna.bello@uniroma3.it; 2 Department of Psychology of Social and Developmental Processes, Sapienza University of Rome, 00185 Roma, Italy; fabio.presaghi@uniroma1.it; 3 Department of Neuroscience, Imaging and Clinical Sciences, University G. D’Annunzio Chieti-Pescara, 66100 Chieti, Italy; tiziana.aureli@unich.it

**Keywords:** pointing gesture, word comprehension and production, developmental trajectories, MB-CDI, infancy, individual differences, moderating effects, multilevel modelling

## Abstract

The present study investigated the moderating role of early communicative pointing on the developmental trends of word comprehension and production over the second year of life. Seventy-seven infants were involved in an experimental pointing task (T-POINT) in sessions at 9 and 12 months, and the MB-CDI questionnaire was filled in by their parents at 15, 18 and 24 months. Based on the age at which the infants were seen to use pointing, they were classified into three groups: the ‘Early’ pointers, who first pointed during the 9-month session; the ‘Typical’ pointers, who first pointed in the 12-month session; and the ‘Late’ pointers, who never pointed in either of the sessions. Using multilevel modelling, we traced the developmental trajectories and individual differences for the two lexical domains of word comprehension and production according to the three pointing groups. The main results showed that compared to the Typical pointers: (i) the Early pointers were faster for word comprehension development, and were similar for word production; (ii) the Late pointers showed lexical delay before 18 months for word comprehension, and between 18 and 24 months for word production. These data are discussed in light of the different roles of early pointing on receptive compared to expressive vocabulary development.

## 1. Introduction

A traditional theme in infant research is the association between gesture and first language development, and more specifically between pointing and lexical acquisition [1,2]. Empirical data have consistently confirmed this relation as concurrent and predictive [3]. However, a full understanding of how pointing impacts on the longitudinal progression of lexical acquisition has yet to be achieved. In particular, whether the impact would be similar for word comprehension and production, deserves to be considered. According to literature, the two domains follow different developmental processes: the first words are comprehended at around 8 months, whereas they are produced at around 12 months; moreover, word comprehension ability increases linearly from 12 to 18 months, with significant interindividual differences occurring mostly at earlier ages, whereas word production develops rapidly at around the middle of the second year, with individual differences between children being much wider from age 2;0 to 3;0 [4,5,6,7]. Differences between the two domains in pointing-language relationships have already been found by research. However, how these differences interact with the advancing of the ability to comprehend and produce words over the first years of life has not been examined so far.

### Pointing-Language Relationship

Research has shown that infants begin to use pointing as a communicative tool to signal different intentions—for requesting, declaring or informing—at around their first birthday; however, there have been discrepancies between studies with respect to the mean age at which infants first use pointing. Depending on the research design, this measure has been shown to vary from 10 months and 29 days in a parental report study [8], to 12 months and 3 days in an experimental study [9], to 12 months and 20 days in a naturalistic study [10]. In general, research found that around 90% of typically developing infants start pointing between 9 and 13 months of age [8], or between 10 and 14 months of age [9], and that all typically developing children do so before 18 months [10]. Based on these data, individual differences are assumed to occur in the emergence of pointing, with early onset of pointing appearing at around 9 months, typical onset of pointing at around 12 months, and late emergence of pointing after 13 months.

The communicative power of pointing is made stronger by the multimodal use of the gesture. Very early after pointing onset, infants couple the gesture with vocalisations, and some months later, with words [11,12,13]. The strict association between pointing and language as two integrated components of a unitary communicative system [14] is confirmed by the evidence of pointing as a predictive signal of the language capacity that emerges some months later [3]: the more the pointing gesture is used by children, the higher their level of language ability, and this relationship becomes stronger with age. As Iverson and Goldin-Meadow [15] indicated, pointing (and other deictic gestures) precedes language and has a facilitating role in early language development, as it “allows children to communicate meanings that they may have difficulty expressing verbally” (p. 367). 

To deepen the understanding of the predictive gesture–language relationship, the handful of studies that have considered the effects of early pointing production on word comprehension and/or word production in the second year of life have shown consistent results. Desrochers and colleagues [16] showed that infants who were “precocious” in their production of pointing (i.e., using pointing before 13 months) obtained higher total scores than their “late” peers for both expressive and receptive language, as measured by the Reynell Developmental Language Scale test at 24 months. Brooks and Meltzoff [17] reported that infants who pointed at 10 to 11 months had faster growth of their productive vocabulary in their second year. Regression analysis showed that pointing production significantly improved the growth curve for word production beyond the age (of pointing) alone, which explained 4% of the variance. Lüke and colleagues [18] showed that index-finger pointing frequency at 12 months explained 17% and 21% of the variance for word comprehension and production when measured at 24 months. Moreover, pointing at 12 months predicted better morphological and syntactic skills at 24 months [18], and also language skills (i.e., sentence comprehension and repetition, word and grammar production) at 5 and 6 years [19]. 

To support the importance of early pointing, research has shown that children who are late in language acquisition show a delay or impairment in pointing between 12 and 18 months of life, which suggests that late pointing can be considered an early common marker of possible language delay [18,20,21]. The specific predictive role of the onset of pointing on lexical development was investigated by McGillion and colleagues [6] using regression models. They showed that the onset age of pointing in a naturalistic study predicted word comprehension at 18 months, but did not predict the onset of the first words or word production at 18 months; these were instead both predicted by phonological readiness (i.e., ‘babbling’). 

To sum up, pointing production around the first year predicts the typical vocabulary development at the end of the second year, and even at 5 to 6 years of age. In addition, measurement of the frequency of pointing in the second year showed that this predictive relationship was evident also in children with language delay [19,21]. However, no studies have looked at how the progression of vocabulary growth—both receptive and expressive—is influenced by the onset age of pointing; i.e., how the onset of pointing interacts with the passing age in terms of shaping the developmental trend of vocabulary acquisition. This is of importance, because it would enable research into possible differences between early and late pointers in this process, to thus contribute to a more detailed view of the developmental trajectories of these skills and the individual differences. 

## 2. The Present Study

The present study was designed to add to research on the pointing–language relationship by examining the moderating role of early pointing on the growth of the infant lexical skills. According to previous research, the pointing gesture is produced with different hand shapes at its emergence, but only the typical index-finger pointing—compared to whole-hand pointing—is considered as a valid signal of the infant communicative intentionality [22,23]. Moreover, the gaze given to other people while pointing is believed to be the most powerful evidence of the gestural communicative intent [8,24]. As the expected predictive role of pointing in language acquisition is grounded on the communicative nature of the gesture, we planned to observe the pointing production in our laboratory, under the controlled conditions provided by the experimental sessions, rather than in the home, in a naturalistic, free setting. In addition, we focused on the age at which infants first pointed in our laboratory, instead of the frequencies of pointing, because we were interested in the infant capacity to engage in this behaviour rather than on its use. For the lexical skills, we collected the infant data using parental reports, using a widely validated instrument to measure this ability. 

To fulfil the aim of the study, a longitudinal research design was used, which included two age points (i.e., 9 and 12 months) for collection of the gestural data, and three age points (i.e., 15, 18 and 24 months) for the collection of the linguistic data. Based on previous studies that have shown a predictive relationship between pointing onset and vocabulary use [3], we hypothesised that the age at which infants were observed to first point in our laboratory would show an interaction with the age for the shaping of the infant lexical development over the second year of life. In particular, infants who pointed early (i.e., at 9 months) were expected to improve word comprehension at a higher rate than infants who first pointed at the typical age (i.e., 12 months). The same was expected for word production, although the onset of pointing has not emerged as a determinant for the initiation of this ability [6]. We also expected that infants who pointed at neither age would improve both word comprehension and word production at a slower rate than infants who first pointed at the typical age.

To trace the developmental trajectories of word comprehension and production, a multilevel modelling technique was applied to our longitudinal data, with the age of the first observed pointing included in the model to find the moderating effects of this variable on both trajectories. This method allows the variance at both the group level and the subject level to be taken into account, and thus allows to estimate the developmental curves that are common to all infants (i.e., the normative trend), and the developmental curves specific for each infant (i.e., the individual differences). Moreover, to investigate how the developmental curves were affected by the onset age of the pointing gesture, we compared early pointers to typical pointers, and late pointers to typical pointers. 

## 3. Materials and Methods

### 3.1. Participants

Seventy-seven infants (40 girls) were observed in a laboratory to measure infant pointing production at 9 and 12 months of age (range, 7 days before or after their birthday). At 15, 18 and 24 months of infant age (range, 7 days before or after their birthday), the mothers filled in the Italian adaptation of the McArthur-Bates Communicative Development Inventories (MB-CDI) [5,25]. Out of the full sample, data were collected on communicative–linguistic development for 69 infants at 15 months, 64 at 18 months, and 53 at 24 months. 

The sample included full-term infants born in the local hospital, living in two middle-sized cities of central Italy. Cases of prematurity and bilingualism were excluded. All infants were healthy and typically developing. They came from two-parent families of middle and middle-high socio-economic status (as determined by parental educational level). 

All of the parents agreed to participate after receiving clear and full information about the study. The study met the ethical guidelines for human subject protection, including adherence to the legal requirements of the country (Declaration of Helsinki). 

### 3.2. Materials and Procedures 

#### 3.2.1. T-POINT

The infants were involved in an experimental pointing task [8] that was designed to elicit imperative and declarative pointing gestures in comprehension and in production. In the present study, we followed the version of the task used by Aureli and colleagues [26] (indicated as the T-POINT task). Only the condition aimed to assess pointing production has been considered for the present analysis.

The infants were tested individually in a playroom equipped with a one-way mirror, and they were videotaped using two cameras. Before the testing phase, each child participated with the parent in a familiarisation phase in a room next to the laboratory, where there were some toys available. The T-POINT comprises two experimental conditions that involve the infant, the experimenter and a proximal or distal stimulus under imperative and declarative conditions, respectively. The proximal stimuli used were two sets of maracas and a wind-up toy airplane. The distal stimuli used were a mobile of Winnie the Pooh and flashing Christmas lights. The conditions consisted of three trials (for a total of six trials), with each presented in a random order. Each trial lasted around 1.5 min. The infant sat on the parent’s legs at a table in front of the experimenter, a woman. 

In each of the two conditions, the experimenter first made eye contact with the child while the stimulus was activated. Then, the experimenter looked silently at the child waiting for the child’s reaction. Then, she gave the toy to the child in the imperative condition, or looked at and named the stimulus in the declarative condition for a more detailed description of the procedure, see [11,26].

All of the infant behaviours related to the stimuli were coded from the videotapes using the computer-based observation software, INTERACT. Only those trials in which the stimuli attracted the child’s attention were coded. Here, we took into consideration the pointing gestures, defined as arm and index-finger extension in the direction of the stimulus (without touching it), while the remaining fingers were curled lightly or tightly under the hand [24]. Only pointing gestures accompanied by a gaze at the experimenter—within a 2 s window before/after the pointing gesture, or during it—were counted, because a gaze is a powerful signal of the communicative intention of an infant [8,24]. As the intention of pointing was not considered in the present study, we summed up the number of pointing gestures under the two conditions (imperative and declarative).

Four trained graduate students coded all of the sessions. Two of the authors (PP, TA) coded 20% of the sessions and obtained values of K Cohen not less than 0.90 under each condition at each age. 

As indicated above, only index-finger pointing that was accompanied by a gaze at the experimenter was analysed in the present study, as this is considered the most correct signal of the infant’s communicative intention. Since no differences in the frequency of pointing between the declarative and imperative intention from 9 to 18 months of age emerged in a previous study [13], no hypothesis was formulated on pointing-language relationship with respect to the intention of the pointing; therefore, we considered the age at which the infants were observed to first point, irrespective of the intention that would cause the infant to point. 

#### 3.2.2. MB-CDI

The Italian version of the MB-CDI [5] comprises two forms: Words and Gestures (WG) and Words and Sentences (WS). The WG form has been validated for children of 8–24 months of age, and the WS form has been validated for children of 18–36 months of age.

The WG inventory includes a vocabulary checklist that consists of 408 words. The parent was asked to indicate if the child understood and produced each word on the checklist. A score of 1 was given for each item checked (word comprehended, word produced). The total receptive and expressive vocabulary scores corresponded to the sum of items marked as comprehended and produced, respectively.

The WS inventory includes a checklist of 670 items. For each item, the parents reported whether the child produced the word. The total expressive vocabulary score corresponded to the sum of the items marked as produced. 

### 3.3. Analysis Plan

The main aim of the present longitudinal study was to investigate the role of pointing on the development of early language acquisition. For this, multilevel polynomial models were implemented, which allowed the trajectories of both word comprehension and production to be traced, as well as the effects of pointing on these trajectories. In longitudinal studies which include nested observations within each infant, multilevel modelling allows to disentangle individual differences from the normative trend by relaxing assumptions of Ordinary Least Squares methods such as equally spaced observations, missing at random data and homoschedasticity. The direct effects of pointing on comprehension and production were also analysed. 

As the word checklists used in both the MB-CDI forms to assess the infant word comprehension and production skills were different based on their age, the respective proportions of comprehended and produced words on the total number of words included in the MB-CDI check-lists were computed and used as dependent variables. For the measure of pointing, the age at which the infants pointed for the first time in this experimental setting was considered. Based on this criterion, three groups of pointers were defined: the Early, Typical and Late pointers. These were defined by children who were observed to first point at 9 months or 12 months, or not to use pointing in either session, respectively. This classification was used as the variable to test the direct effects of pointing on mean word comprehension and production, and the moderating effects of pointing on the developmental trend of the proportion of word comprehension at 15 and 18 months, and the proportion of word production at 15, 18 and 24 months. 

To investigate pointing effects on vocabulary acquisition, a binomial multilevel regression model was run [27]. The developmental trend of the proportion of word comprehension at 15 and 18 months, and the proportion of word production at 15, 18 and 24 months, were predicted by fixed effects—linear for comprehension, and linear and quadratic for production—of Age, Gender (girls) and age of first observed pointing. The moderating effects of pointing on the two trends (i.e., comprehension, production) were tested by including the pointing measure in the model. As the measure of pointing had three modalities (i.e., Early vs. Typical vs. Late), the variable was decomposed into two dichotomous variables where the first compared the Typical vs. Early pointers; and the second compared the Typical vs. Late pointers. The random effects among the infants were also estimated for the age trend, to allow for individual differences in the variability of the proportions of word comprehension and word production. The data were fitted to a two-level (infants as level 2; observations as level 1) random intercept model (i.e., allowing individual differences of the infants to be estimated only at the beginning of the observations). The general multilevel models used were as given in Equations (1)–(4):

Fixed effect models:

Logit (Comprehension πij) = β0ij + β1j linear trend + β2 Gender (girls) + β3 Early pointer + β4 Late pointer + β5 linear trend × Early pointer + β6 linear trend × Late pointer(1)

Logit(Production πij) = β0ij + β1j linear trend + β2 quadratic trend + β3 Gender (girls) + β4 Early pointer + β5 Late pointer + β6 linear trend×Early pointer + β7 linear trend × Late pointer + β8 quadratic trend × Early pointer + β9 quadratic trend × Late pointer(2)

Random effects model (common to both fixed effects models):

β0j = β0 + u0j + e0i(3)

β1j = β1 + u1j(4)

where πij is the probability (proportion) of infant j word comprehension (or production) at the *i*-th observation. Individual differences among the infants at the beginning (u0j), on the linear trend (u1j), or in the covariance between individual differences at the beginning of observation and the individual differences in the linear trend (u01j) were estimated as, respectively, the variances (σ^2^_0j_, σ^2^_1j_) and covariance (σ_01j_) parameters. All of the continuous predictors were centred around the overall average of the sample, to ease the interpretation of moderating effects.

All of the models were estimated using Markov Chain Monte Carlo (MCMC) estimation methods, with an initial burning of 500, followed by a number of monitoring simulations that ranged from 40,000 to 60,000; the starting values were provided using maximum likelihood methods [24]. Better estimates were obtained using MCMC estimation methods, given that the number of units was not high. To test whether a regression coefficient or a random parameter (either variance or covariance) was significantly different from zero, the 95% Bayesian Credible Interval (95% Cred. I.) obtained from MCMC estimates were considered [24]. The MlwiN statistical software was used for estimation of all of the effects [27]. A parameter was considered significantly different from zero when the 95% Cred. I. did not include the zero value.

## 4. Results

### 4.1. Descriptive Analysis

On the basis of the first time at which the infants pointed in the experimental sessions, three comparison groups were defined: the Early pointers (N = 17; girls, 47%), the Typical pointers (N = 36; girls, 44%) and the Late pointers (N = 24; girls, 67%), as the infants who first pointed at 9 or 12 months, or at neither age, respectively. Table 1 gives the descriptive statistics for the frequencies of word comprehension at 15 and 18 months and word production at 15, 18 and 24 months as functions of the three groups. Considering the total mean frequencies, at 15 months, infants comprehended around 51% of the MC-CDI list, and their ability consolidated between 15 and 18 months (reaching 61% of the word list). On the contrary, at 15 months, word production was emergent (number of words, ~6%), increasing between 15 and 18 months—where they reached 17% of the word list—and then at the end of the period considered they produced around 48% of the list. Our descriptive data thus confirmed that word comprehension precedes word production during the second year of life. The standard deviations were higher for production than comprehension. 

Comparing the three groups (i.e., the Typical, Early, Late pointers), clear differences appeared in their word comprehension and word production skills. For comprehension, infants who showed the first observed pointing at 9 months (i.e., the Early pointers) showed greater vocabulary use at 15 and 18 months, compared to the other two groups. 

The Late pointers showed a similar vocabulary size at 15 and 18 months with respect to the Typical pointers. At 15 and 18 months, the three groups were relatively similar for word production, which was very low. The increase in word production between 18 and 24 months was different across the three groups: the Early pointers showed a greater increase compared to the other two groups, and the Late pointers exceeded the Typical pointers in terms of the increase.

### 4.2. Main Analysis

We estimated the linear and quadratic developmental trends of word comprehension and word production according to the fixed effects of Age, Gender and age of the first observed pointing. Then, our hypothesis was tested by estimation of the moderating effects of the first observed pointing on these trends, at both the group and the individual levels (Table 2). 

#### 4.2.1. Word Comprehension

The linear trend for word comprehension was significant and positive (b = 0.109; 95% Bayesian Cred. I.: 0.002; 0.219), and girls showed significantly higher levels of comprehension than boys (b = 0.415; 95% Bayesian Cred. I.: 0.004; 0.787). The Early pointers showed similarly higher levels of proportion of mean word comprehension than the Typical pointers (Early pointers: b = 0.219; 95% Bayesian Cred. I.: −0.261; 0.636). Moreover, the Late pointers showed similarly lower levels of comprehension than the Typical pointers (b = −0.174; 95% Bayesian Cred. I.: −0.602; 0.271). 

**Figure 1:**
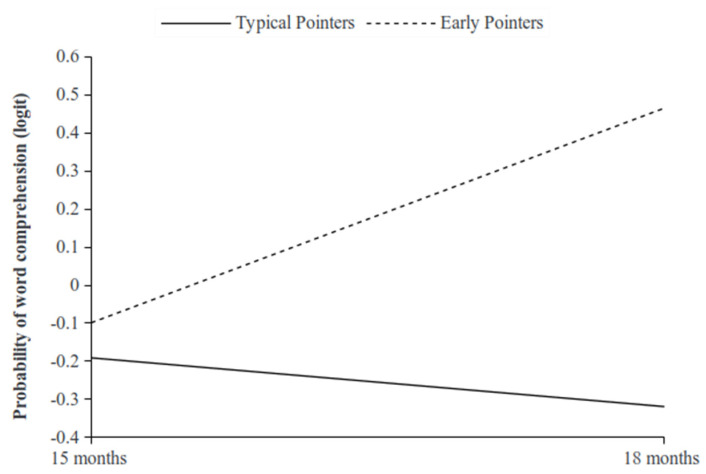
Simple slopes for the moderating effect of the Early pointers (vs. the Typical pointers) on the linear trend of the proportion of word comprehension.

**Figure 2:**
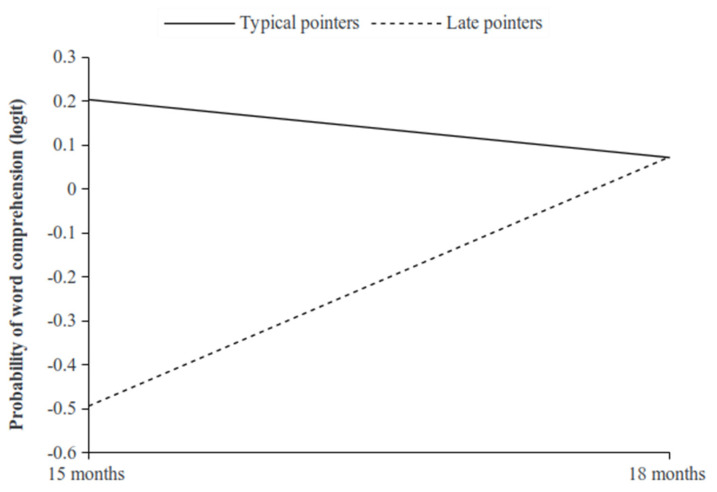
Simple slopes for the moderating effect of the Late pointers (vs. the Typical pointers) on the linear trend of the proportion of word comprehension.

Turning to the moderating effects, those for the Early pointers on the linear developmental trend of comprehension were positive with respect to the Typical pointers, as expected, and nearly reached significance (b = 0.173; 95% Bayesian Cred. I.: −0.014; 0.369). The moderating effects of the Late pointers on the linear trend of comprehension with respect to the Typical pointers were positive and significant (b = 0.175; 95% Bayesian Cred. I.: 0.014; 0.375). These effects are shown in Figure 1 and Figure 2, respectively. 

Further, the Early pointers developmental trend was always above that of the Typical pointers, and increased over time with respect to the Typical pointers trend, which appeared to remain flat over the 15 to 18 months period (Figure 1). On the contrary, the Late pointers developmental trend was below that of the Typical pointers (Figure 2), although it increased over time to that reached by the Typical pointers at the end of 18 months. To sum up, as hypothesised, both the Early pointers and the Late pointers showed growth trends in word comprehension, whereby the Early pointers showed a higher rate of comprehension than the Late pointers and also the Typical pointers. 

Finally, independent of the pointer groups, the infants differed significantly among themselves at the beginning of the observations (σ^2^_0j_ = 0.666; 95% Bayesian Cred. I.: 0.473; 0.934) as well as in the linear trends (σ^2^_1j_ = 0.080; 95% Bayesian Cred. I.: 0.053; 0.118) (Table 2). These differences remained stable over the period, as no significant covariation between individual differences at the beginning of the observations and individual differences in the linear trends were seen (σ_01j_ = 0.044; 95% Bayesian Cred. I.: −0.016; 0.113). This means that the children differed in the proportions of words understood at the beginning of the observations and in their developmental trends, and these differences were constant across the observations.

#### 4.2.2. Word Production

With respect to word production, as given in Table 2, a significant and strongly increasing linear trend was seen (b = 0.543; 95% Bayesian Cred. I.: 0.422; 0.673), while the quadratic trend was significant and slightly decreasing (b = −0.025; 95% Bayesian Cred. I.: −0.031; −0.020), whereby the increase was faster between 15 and 18 months than between 18 and 24 months. Girls did not show differences from boys here (b = 0.084; 95% Bayesian Cred. I.: −0.400; 0.495). The Early pointers proportion of mean words was lower than that of the Typical pointers, although this difference did not reach significance (b = −0.518; 95% Bayesian Cred. I.: −1.062; 0.021). Also, word production for the Late pointers was lower, although not significantly so, than that of the Typical pointers (b = −0.482; 95% Bayesian Cred. I.: −1.030; 0.029). 

**Figure 3:**
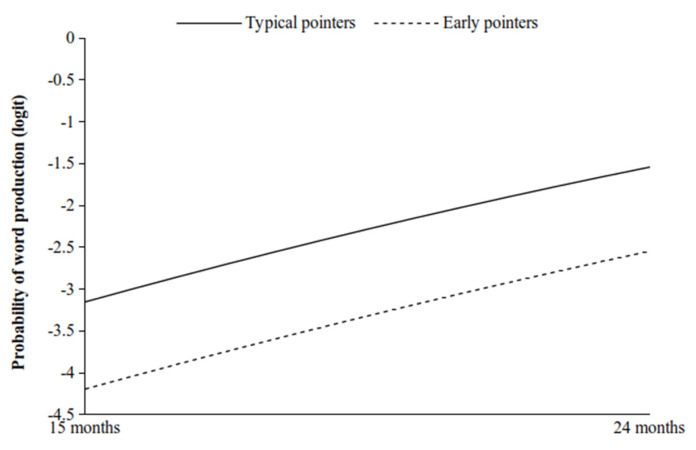
Simple slopes for the moderating effect of the Early pointers (vs. the Typical pointers) on the quadratic trend of the proportion of word production.

**Figure 4:**
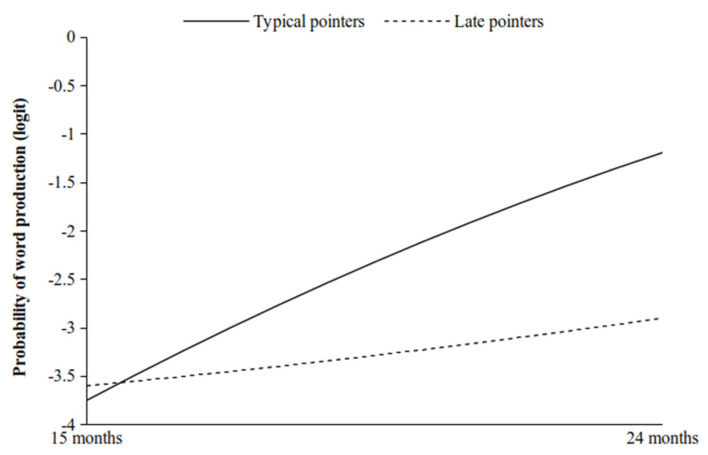
Simple slopes for the moderating effect of the Late pointers (vs. the Typical pointers) on the quadratic trend of the proportion of word production.

The moderating effects of the Early pointers compared to the Typical pointers on both the linear and quadratic trends were not significant (linear: b = 0.006; 95% Bayesian Cred. I.: −0.209; 0.194; quadratic: b = 0.003; 95% Bayesian Cred. I.: −0.005; 0.012). In particular, as shown in Figure 3, and contrary to our expectations, the developmental trends of both the Early pointers and the Typical pointers were positively inclined and parallel throughout all of the observations. Finally, the moderating effects of the Late pointers with respect to the Typical pointers on the linear and quadratic trends were both significant (linear: b = −0.310; 95% Bayesian Cred. I.: −0.434; −0.183; quadratic: b = 0.041; 95% Bayesian Cred. I.: 0.032; 0.050). As hypothesised, the simple slope analysis showed that the Late pointers quadratic trend increased at a very slow rate during the observational period, while that of the Typical pointers increased at a higher rate (Figure 4). Therefore, while at 15 months the Late pointers and the Typical pointers showed similar levels of word production, at 24 months the Typical pointers reached a higher level than the Late pointers.

Finally, there were significant individual differences at the first observations among the infants (σ^2^_0j_ = 1.115; 95% Bayesian Cred. I.: 0.778; 1.587) and for the rate of linear growth (σ^2^_1j_ = 0.061; 95% Bayesian Cred. I.: 0.041; 0.090), although the two random effects did not show significant covariance (σ_01j_ = −0.004; 95% Bayesian Cred. I.: −0.077; 0.068). This indicates that the children differed in the proportions of words produced at the beginning and in their developmental trends, and that these differences remained constant across all of the observations.

## 5. Discussion

The main aim of the present study was to analyse the moderating effects of communicative pointing on lexical acquisition in the second year of life. In particular, we hypothesised that early index-finger pointing would positively influence the growth curve of word comprehension and production, while late index-finger pointing would be a negative influencing factor. We involved infants in two experimental sessions at 9 and 12 months of life, and focused on the age at which the infants were first observed to point. On the basis of studies that have shown that the age of 11–12 months is typical for pointing acquisition, we defined the Early pointers as the infants who produced a pointing gesture in the first experimental session, when they were 9 months old, and we defined the Late pointers as the infants who did not produce any pointing in the sessions at 9 months or 12 months. We defined the Typical pointers as the infants who produced pointing in the second experimental session, when they were 12 months old.

### 5.1. Moderating Effects of the Pointing Gesture on Word Comprehension and Word Production

As a first result, our data show that pointing in the first session (i.e., the Early pointers) or never pointing in any session (i.e., the Late pointers) had no direct effects on word comprehension and production. Thus, the Early pointers and the Late pointers did not differ in the total number of words comprehended and produced with respect to the Typical pointers. Indeed, they differed in the developmental trends of both word comprehension and production. 

The Early pointers showed a positive trend in word comprehension, which developed at a higher rate than for the Typical pointers, as we had hypothesised. For word production, contrary to our hypothesis, the Early pointers showed a trend that was similar to the Typical pointers. Thus, the acquisition of pointing at an early age facilitates the development of the receptive lexicon, but not the development of the expressive lexicon, in the observed age period. For the Late pointers, at 15 months they understood fewer words compared to the Typical pointers. However, from 15 months onwards, they showed fast growth, such that at 18 months they reached a receptive ability that was similar to that of the Typical pointers. It could be that before 15 months the Late pointers had a slower receptive developmental trend than the Typical pointers, but then they accelerated their trend later on; thus, our hypothesis can be said to be partially confirmed. For word production, the Late pointers showed a very slow rate of development compared to the Typical pointers, as was expected. In summary, compared to the Typical pointers, the Late pointers showed a lexical delay, which occurred before 18 months for word comprehension, while for word production it occurred between 18 and 24 months. 

Overall, our data confirm the data from the literature on the pointing–language relationship [3]. This relationship has been shown to change depending on the age and on different aspects of language, such as prosody, vocabulary, grammar emergence and grammar abilities (e.g., for the relation between pointing and prosody, see [11]; between pointing and vocabulary, see [18]; between pointing and transition from one- to two-word combinations, see [28]; between pointing and grammar abilities, see [19]). The present data add to previous studies by showing that being early in communicative pointing benefits infants in terms of their lexical growing rate, whereas being late is associated with slower lexical development. In more detail, early pointers have an advantage for their word comprehension, while they show word production development similar to typical pointers. This result is in line with research showing that gestural production (as reported by parents) relates to word comprehension more than to word production [7,29]. In particular, this is consistent with McGillion and colleagues [10], who showed that pointing onset detected at home predicted word comprehension at 18 months, but not word production, which was instead predicted by phonological readiness (i.e., babbling). Both of these studies indicate different roles of pointing onset on receptive vocabulary development with respect to expressive vocabulary development. 

As argued in many studies, pointing allows infants to direct attention to an interesting object or event, and also to elicit a comment from the caregiver about the object they are sharing or the event to which they are jointly attending [1,30,31]. According to the authoritative Werner and Kaplan [32] claim, infant pointing is a referential motor act, which, when used in joint attention episodes, creates a context of communicative exchanges, which stimulates the caregiver to name the pointed target [33,34], thus promoting the infant’s language acquisition [35]. While it would follow from these theoretical assumptions that the infant’s pointing production influences both word comprehension and production, our findings and those of McGillion and colleagues [10] appear to question this inference, as they show that pointing production gives early pointers an advantage over their ‘typical’ peers in the word comprehension domain only. 

According to the pragmatic point of view on language acquisition, the association between referent, meaning and label in the context of communicative exchanges is needed to allow language acquisition to develop, whatever the process under scrutiny is, as word comprehension or production. Learning the meaning of words (i.e., word comprehension) requires the infants to associate the new words with the referent by listening to the phonetic and semantic details [36]. For this, labels have to occur frequently in the presence of the referent identified by the pointing gesture, and the infant has to look at it at length [37,38], with the infant attention extended by the concurrent use of pointing.

For the development of word production, the process is more complex and the above conditions are not enough. As McGillion and colleagues [10] revealed, phonological mastering is a determinant for making the words to be pronounced, and this ability is linked to the infant’s use of prelinguistic vocalisations. According to the evidence, the age of babbling onset has been shown to predict the age at which infants are observed to produce their first four words, and strong positive correlation has been shown to link babbling onset to the quality of babbling and expressive lexical growth [39]. Maternal responses also influence word production, as demonstrated by Gros-Louis and colleagues [40]. They showed that vocabulary production at 15 months (evaluated using the MD-CDI) was predicted by the mother’s naming in response to the child’s vocalisations, and that after 11 months of age (i.e., by the time the infant’s vocalisations directed to referents increased significantly), the sensitive mother’s responses to the infant’s vocalisations also increased, and were associated with an increase in developmentally advanced consonant–vowel infant vocalisations. This process with the mother’s responses and infant vocalisations influencing each other in a dynamic manner is favoured by the emergence of the multimodal use of pointing. Whereas before 12 months of age, infant pointing without speech occurs more frequently than gesture–speech combinations, from 15 months onwards, gesture–vocal combinations are more frequent than the production of gestures alone or vocalisations alone [11,12,41]. In particular, from 17 months on, pointing alone is likely to decrease, while pointing–vocal coupling increases rapidly [13]. Indeed, it is precisely these pointing–vocal combinations that foster word production in the communicative context described before [41]. 

How can the picture just described be helpful to explain the lexical development shown by early pointers in our study? At 9 months, this group of infants showed the canonical index-finger pointing (instead of whole-hand pointing) accompanied by a gaze towards the partner, thus showing the gestural behaviour that is believed to involve the understanding of the communicative intention of the gesture [8,23]. In other words, they produced a relatively sophisticated pointing gesture. It might be that with early pointers being effective in communicating through the pointing, they rely on their gesture competence more than their vocal competence, and use gestural effectiveness to induce the adult to name the pointed target, thus promoting their word comprehension ability. Word production might not profit from early pointing as well, as the development of word production requires the ability to refer verbally to the pointed object, thus implying quantitative and qualitative changes in the use of vocalisations [40] that the 9-month-old pointers might not have developed yet. Instead, it might be that the typical pointers who were observed to first point at 12 months can accompany their pointing gestures with vocalisations, thus using a multimodal means of communicating that is a far more powerful signal for the caregiver to improve linguistic exchanges. Through eliciting the attention of and the naming by the caregiver while being able to vocalise when pointing, such typical pointers might be in the most favourable condition for advancing the processes underlying acquisition of both word comprehension and word production. 

Our findings also concern late pointers, who showed delays in both word comprehension and word production. This result which arose from considering the age of the first observed pointing is in line with studies where pointing frequencies have been measured. In particular, such studies have shown that a low rate of pointing at 12 months [18] or at 18 months [21] represents an early common marker for children with language delay, compared to typically linguistic children (in the second and third years of life). Based on the present study, we suggest that a late onset age of pointing is also an early index of infants at risk of language delay. In particular, the late onset of pointing is an index of word comprehension delay at 15 months and word production delay at 18 and 24 months. To sum up, late pointers might also be late talkers, as they showed a delay in word production at 24 months [42,43].

### 5.2. Strengths and Limitations of the Study

The present study provides valuable and new insights into the longstanding issue of pointing–language relationships. In particular, our results contribute to the debate about the impact of pointing onset on lexicon acquisition, by showing that this parameter interacts with age in the second year of life, to shape the developmental trajectories of word comprehension and word production. 

The strengths of the present study include the longitudinal design and the repeated measures; in particular, it is worth noting the implementation of the multilevel modelling technique, which, while analysing the moderating role of pointing, allowed estimation, for the first time, of lexical developmental growth over the second year of life. We have shown here that word comprehension increases with a gradual trend between 15 and 18 months, whereas word production, as well as also showing a significant linear developmental trend, shows a quadratic trend with a steeper slope between 15 and 18 months, compared to between 18 and 24 months. In both cases, we have confirmed a number of cross-sectional studies that have documented a gradual increase in word comprehension during the first half of the second year, and a burst of word production soon after. The present study adds to those studies by modelling the developmental trajectories of language acquisition, instead of only suggesting it [44]. Indeed, we have estimated precisely how word comprehension and production grow with advancing age, instead of simply documenting how much they differ between the ages. In this framework, infants who never pointed at 9 and 12 months were found to have a delayed growth in both domains of language acquisition, which is shown to spread over the entire subsequent age period. We think that this finding could be of importance in the clinical context, to plan intervention programs that could start from the beginning of the second year of life.

Some limitations of the present study need to be considered in the interpretation of the results and as directions of future research. As to the measures, we considered the emergence of pointing in terms of the age at which the infants first pointed in this experimental study. This measure was shown to be valid, as it allowed to identify three groups of infants that differed significantly according to the impact of the first observed pointing on their language development. However, the infant’s age at the observed pointing in a lab might not be the onset age of pointing; we suggest that future studies on the impact of the pointing onset on language developmental trajectories could capture this measure by a diary instrument in a naturalistic setting [45]. We also acknowledge that a more in-depth understanding of pointing–language relationships can consider not only the vocabulary size, as we did, but also the onset age of word comprehension and production. Future research can be focussed in that direction, by including the very emergence of communicative capacity in both the gestural and language domains. Secondly, the data from the present study are limited to two points of assessment for word comprehension (i.e., 15 and 18 months) and three points of assessment for word production (i.e., 15, 18 and 24 months). We can suggest further longitudinal studies that investigate the role of pointing in receptive and expressive vocabulary growth trends also in the third year of life. Thirdly, because of the differences in the roles of pointing for word comprehension and word production shown here, it would be particularly informative to distinguish the pointing gesture without or with vocalisations to verify our hypothesis that early pointers use more pointing without vocalisations than typical pointers. Moreover, we found wide interindividual differences both in pointing and lexical development, which could be related to children’s contextual factors (e.g., parental education, socio-economic status) that unfortunately have not been explored in the present study. Future studies should take into consideration these factors and examine how they affect the relationship between pointing and linguistic acquisition. Finally, since we found that late pointers are delayed in language acquisition until the end of the second year of life, we suggest that to further investigate the pointing as an early index of language delay, future research should be required to measure not only the frequency of pointing, as usual, but also the onset age of pointing and, using this measure, to follow early language development until the assessment of language delay in the third year of life.

## 6. Conclusions

To our knowledge, the present study is the first to show the moderating role of communicative pointing for lexical acquisition trajectories over the second year of life. By applying multilevel modelling to these longitudinal data and including the age of the first observed pointing in the model, we have shown that infants who pointed at an early age in our experimental sessions improved the rate of their word comprehension development, but not that of their word production, whereas late pointers were slower in the increase in both receptive and expressive skills. 

These data contribute to a deepening of previous studies on the relationships between communicative pointing and lexical acquisition, and allow us to argue that the pointing gesture not only pre-dates language development [15], but is also fundamentally tied to it—and predicts it—in different ways at different ages. As we have shown, the three groups of children identified in our study as early, typical and late pointers were shown to achieve lexical skills with trajectories that differed in the rates of their increases. This suggests that language development is made up of communicative achievements that are tied to both individual tendencies and social opportunities from the beginning: when interacting with their partner, some infants can use more vocalisations while others rely more on gestures, which in turn influences how they advance in the development of their abilities to comprehend and produce words. As suggested by Beuker and colleagues [46], development has the form of a matrix, with factors that mutually influence each other. The moderating role of pointing represents a major factor that impacts language development by modifying the rate of its growth, and thus it adds to the influencing factors already defined by previous studies that can make some pathways more probable than others.

## Figures and Tables

**Table 1:** Word comprehension and word production descriptive statistics for frequencies of the Typical, Early and Late pointers.

	Typical-Pointers			Early-Pointers			Late-Pointers			Total		
	Mean	SD	Min-Max	Mean	SD	Min-Max	Mean	SD	Min-Max	Mean	SD	Min-Max
*Word Comprehension* ^a^												
15 months	213.0	72.9	70; 341	226.7	55.7	127; 367	189.7	81.8	81; 357	208.3	73.2	70; 367
18 months	230.9	94.3	79; 373	293.7	56.8	181; 369	249.3	94.2	114; 407	250.7	89.5	79; 407
*Word Production* ^b^												
15 months	27.9	36.1	3; 182	19.8	16.2	2; 61	17.8	12.1	1; 43	22.9	26.6	1; 182
18 months	89.7	90.3	12; 314	69.1	63.1	0; 188	43.3	46.7	1; 204	70.4	74.6	0; 314
24 months	296.5	147.2	15; 541	390.5	202.8	30; 623	310.5	207.4	15; 655	324.0	166.5	15; 655

Note: ^a^ Maximum number of words in the MB-CDI check list is 408. ^b^ Maximum number of words in the MB-CDI check list is 408 at 15 and 18 months, and 670 at 24 months.

**Table 2:** Markov Chain Monte Carlo estimates for trend effects and moderating effects of pointing (categorical: 0 = Typical pointers; 1 = Early pointers; 2 = Late pointers) on word comprehension and word production from 15 to 24 months.

Model	Comprehension ^a^			Production ^b^		
	b	s.e.	95% Bayes. Cred. I.	b	s.e.	95% Bayes. Cred. I.
*Fixed effects*						
Constant	−0.036	0.144	−0.284; 0.277	−2.802	0.189	−3.160; −2.44
Linear trend (months-gm)	0.109	0.056	0.002; 0.219	0.543	0.059	0.422; 0.673
Quadratic trend (months-gm^2^) ^c^	--	--	--	−0.025	0.003	−0.031; −0.020
Gender (girls)	0.415	0.193	0.004; 0.787	0.084	0.230	−0.400; 0.495
Early pointers	0.219	0.229	−0.261; 0.636	−0.518	0.275	−1.062; 0.021
Late pointers	−0.174	0.221	−0.602; 0.271	−0.482	0.272	−1.030; 0.029
Linear trend × Early pointers	0.173	0.100	−0.014; 0.369	0.006	0.103	−0.209; 0.194
Quadratic trend × Early pointers ^a^	--	--	--	0.003	0.004	−0.005; 0.012
Linear trend × Late pointers	0.175	0.085	0.014; 0.375	−0.310	0.067	−0.434; −0.183
Quadratic trend × Late pointers ^a^	--	--	--	0.041	0.004	0.032; 0.050
*L-2 Random effects (individual difference among infants)*						
Constant: σ^2^_0j_	0.666	0.119	0.473; 0.934	1.115	0.208	0.778; 1.587
Constant × Linear trend: σ_01j_	0.044	0.033	−0.016; 0.113	−0.004	0.037	−0.077; 0.068
Linear trend: σ^2^_1j_	0.080	0.017	0.053; 0.118	0.061	0.013	0.041; 0.090
Units: id	76			77		
Units: months	133			191		

Note: ^a^ MCMC estimation with 40,000 draws; ^b^ MCMC estimation with 60,000 draws; ^c^ not available for word comprehension.

## Data Availability

The datasets used and analysed in the current study are available from the corrisponding author upon reasonable request.
